# Reproduction of the long-spined sea urchin *Diadema setosum* in the Gulf of Aqaba - implications for the use of gonad-indexes

**DOI:** 10.1038/srep29569

**Published:** 2016-07-12

**Authors:** Omri Bronstein, Andreas Kroh, Yossi Loya

**Affiliations:** 1 Natural History Museum Vienna, Geological-Paleontological Department, Burgring 7, 1010 Vienna, Austria; 2 The Steinhardt Museum of Natural History, Israel National Center for Biodiversity Studies, Tel Aviv University, Tel Aviv 69978, Israel; 3 Department of Zoology, The George S. Wise Faculty of Life Sciences, Tel-Aviv University, Tel-Aviv 69978, Israel

## Abstract

As global warming and climate-change proceeds ever more rapidly, organisms depending on seasonal cues to synchronize reproduction face an unclear future. Reproduction in *Diadema setosum* in the Gulf of Aqaba (Red Sea) is seasonal, with mature individuals occurring from July to October. Gonad indexes (GI), in contrast, indicate that spawning occurs from August through December and suggests two main spawning events. Histological analysis, however, indicate that the second peak of GI values cannot be related to spawning, but rather correspond to recovering individuals. In *Diadema*, examination of GI values alone may thus lead to erroneous conclusions. GI was moderately-strong positively correlated with sea-surface temperatures, but not with chlorophyll-a concentrations or photoperiod. Spawning coincides with the onset of the annual chlorophyll-a increase, however, which might be advantageous for nutrition of the developing larvae. First significant GI increase coincides with the shortening of day-length, which may act as a cue for *D. setosum* gametogenesis. Gametogenesis is highly synchronised between sexes, although the mature phase of females exceeds that of males. The non-complete overlap may represent sampling bias or represent an adaptive strategy for enhancing fertilisation success. Skewed sex ratios (♀:♂ 1:0.59, n = 360) in the Gulf of Aqaba may be related to pollution.

Sea urchins from the genus *Diadema* are some of the most widespread, abundant and ecologically important echinoids in tropical regions^1^. Eight named extant species of *Diadema* are currently recognised^2^,^3^. Some of these contain distinct mitochondrial lineages^4^, which may imply the existence of additional, yet unnamed, species. They inhabit tropical waters from the intertidal zone to a depth of 360 m^5^. Species of *Diadema* are conspicuous members of benthic communities^1^ and are often regarded as keystone species in coral-reef environments^6^.

*Diadema*, like most echinoids, are broadcast spawners, releasing gametes into the seawater^1^. Successful reproduction, therefore, necessitates synchronous spawning of a certain number of individuals within a population to achieve the sperm concentrations needed for fertilisation. As such, population densities as well as reproductive behaviour, such as adult aggregations during spawning, play a vital role in fertilisation success^7^. There are, however, vast spatial and temporal variations in spawning synchrony and aggregative behaviour between different populations, even within a single species – *Diadema antillarum* for example was observed to form tight spawning aggregations in some areas^8^, but not in others^9^. In *Diadema*, as in most other sea urchins, gametogenesis typically follows a set of sequential maturation stages that are characterised by seasonal changes in gonad development and mass^10^. As fertilisation success is highly dependent on intra-population synchronisation, both gamete synthesis and spawning activation are believed to be mediated by external environmental cues^11^. Multiple environmental factors may be involved in regulation of this process and some have been suggested to also play an important role in regulating *Diadema* reproduction; among these are temperature^12^, photoperiod^13^, tides^14^, food availability^15^ and lunar cycles^16^. In addition, the length of the reproductive period has been observed to vary within the geographic range of wide-spread echinoderm species, with populations near the equator spawning more or less continuously and seasonally at the edges of their ranges^12^.

*Diadema setosum* inhabits a vast geographic range^4^, ranging from east Africa and the Red Sea, throughout the entire tropical Indian Ocean to Japan and the South Pacific Islands (Fig. 1). However, in contrast to the extensively studied *D. antillarum*, which has been studied across most of its geographic range, comparatively fewer detailed publications are available on the reproductive cycle of *D. setosum*. Most of the available information on the reproduction of *D. setosum* derives from a major study by John Pearse^12^, who reviewed reproductive patterns of four Info-Pacific echinoderm species based on published and novel fieldwork data. Since then additional accounts on the reproductive cycles of *D. setosum* from selected localities have been published^14^,^17^,^18^. The broad latitudinal range of *Diadema setosum* facilitates an examination of the ‘equatorial model’, one of the most prevalent paradigms in marine invertebrate reproduction. This model predicts that for species with a broad latitudinal distribution, continuous reproduction is expected in the tropics, while a restricted breeding season is expected at higher latitudes^19^. In the Gulf of Aqaba (GOA) *D. setosum* is a major component of the benthic fauna in both coral-reef and rocky environments^20^,^21^, being one of the most abundant echinoderms in these habitats and an important ecosystem engineer.

The GOA is a narrow, semi-enclosed gulf located at the northeast tip of the Red Sea. It is of special research interest since it differs in its environmental parameters from both the Red Sea Main Basin (RSMB) and the Gulf of Suez (GOS) and has been suggested to have acted as a refugium for the Red Sea fauna during Pleistocene sea-level low-stands and salinity crises^22^. The GOA comprises a wide range of different habitats including lagoons, sea grass meadows, mangrove stands and some of the northern-most tropical coral reefs^23^. In contrast to the long and shallow GOS (250 km long and ca. 35 m in average depth), the GOA is shorter and deeper (160 km long and ca. 650 m average depth) and shows different environmental conditions^24^. Despite of their latitudinal similarity and in contrast to the relatively stable conditions of the GOA, the shallow depth of the GOS causes it to be temperate in character, reflecting seasonal changes of the same magnitude as the eastern Mediterranean^25^,^26^. Furthermore, based on species distribution and abundance, James and Pearse^20^ concluded that the environmental conditions within the GOA are more similar to those in the RSMB than to those in the GOS, and that the major faunistic differences between these two gulfs are most likely related to their differing depths. Likewise, the reproductive cycle of some marine invertebrates, including some echinoid species, seem to differ between the two gulfs and the RSMB despite their relative geographical proximity (e.g., *Echinometra* sp.^27^). However, while the reproductive cycle of *D. setosum* is well studied for the GOS^10^,^12^ and the RSMB^28^, no data are so far available for the GOA.

Here we provide the first report on the reproductive biology of *D. setosum* from the GOA with the aim of evaluating the environmental cues that control and synchronise gamete maturation and identify spawning periodicities in this species. In addition, we provide information on sex ratios in naturally occurring *Diadema* populations from two contrasting environments with (a) high (GOA) and (b) low (Zanzibar) levels of anthropogenic interaction and evaluate sexual differences in resource allocation between males and females in these environments.

## Results

### Sex ratios and body size

The sex ratios of *Diadema setosum* from the GOA deviated significantly from a ratio of 1:1 indicating that females are more abundant than males (96 males, 164 females; Chi-square test, *x*^2^ = 17.785, *df* = 1, p < 0.0001), in contrast to the equal ratio recorded in Zanzibar (199 males, 215 females; Chi-square test, *x*^2^ = 0.62, *df *= 1, p = 0.432). No hermaphrodites were observed in any of the samples.

The weights and diameters of *D. setosum* from the GOA and Zanzibar were positively and highly correlated (r^2^ = 0.85, p < 2.2e-16 for the GOA and r^2^ = 0.83, p < 2.2e-16 for Zanzibar), demonstrating allometric weight increase with growth; the linear regression lines of the log transformed data are shown in Fig. 2a–c. The best fit of test diameter to the model was superior to that of test height (r^2^ = 0.82 and r^2^ = 0.73, respectively). No significant differences were observed between the regressions of males and females at all sites.

### Gametogenic cycle

#### Histological inference

Four different gonad maturation stages have been recognised for male and female *D. setosum* (Fig. 3). These stages, termed *Spent*, *Recovering*, *Growing* and *Mature*, (corresponding to stage numbers I to IV, respectively; see Fig. 3 for details), appeared successively throughout the duration of the study from January through December. Despite being generally consistent, some variation among individuals from the same monthly sample was occasionally observed. Gonads maturation stages developed from a gamete and nutritive phagocyte (NP) depleted phase (*Spent*) to a stage of energy replenishment through NPs renewal (*Recovering*), followed by the synthesis of new germinal cells (*Growing*) and the accumulation of ripe gametes (*Mature*) ultimately leading to spawning.

The relative monthly frequencies of the four maturation stages are shown in Fig. 4. Males and females appeared to be highly synchronised with females showing a somewhat extended mature stage in comparison to males. Spent males and females were present in low proportions from late summer through winter (end of August to February). Males at the *Recovering* stage were only evident during winter (November–April), while recovering females were present year-round (although more common in winter). Gametogenesis initiated in May in both males and females and high proportions of growing individuals were present through August in females and as late as October in males. Maturation and spawning occurred from midsummer to early winter (July to October). Mature males and females were first observed in July, however, the duration of the mature female stage was longer and lasted to October, reaching a peak in September.

#### Inference of gonadosomatic indexes

The gonado-somatic indexes of *D. setosum* from the GOA illustrate an annual cycle (Fig. 5 and Supplementary Fig. S1). As no significant differences in individual mean GIs between males and females were found in any of the sampled months (Mann-Whitney *U* test, all FDR corrected p > 0.05), their data were pooled monthly. Although some differences were observed between the different indexes (Supplementary Fig. S1), as expected from the differences in their underlying allometric measurements, they presented similar trends. The observed monthly differences were confirmed by conducting analyses of covariance (ANCOVA) for all 13 monthly samples for both independent variables (test diameter and total wet body weight) to test for monthly differences in gonad weights. Monthly changes of the allometric exponent β (the regression slopes) of both variables were highly comparable and reflect an annual cycle with similar patterns when using either test diameters or total wet weights (Supplementary Fig. S2). GIs reached an annual peak from mid-summer to early winter showing two peaks (August-September and November-December) interrupted by a sharp drop in October (Fig. 5, Supplementary Fig. S1). However, while the first annual maxima in GI index coincided with the presence of mature gametes, no ripe individuals were observed in histological examinations during the second index maximum in November to December. GI values for all indexes were comparably lower during winter from January to May, after which GI build-up was observed. The GI values in February 2011 were similar to those of February 2010.

Mean GI values varied significantly between months (Kruskal-Wallis test, GI_W:_ H = 93.27, *df *= 12, p < 1.15e-14; GI_D:_ H = 99.77, *df *= 12, p < 6.19e-16; SGI: H = 74.8, *df *= 12, p < 4.01e-11). Monthly mean GI levels had a moderate-strong positive correlation with SST (Spearman rank order coefficient, GI_W_: *r*_s _= 0.82, p < 0.01; GI_D_: *r*_s _= 0.63, p < 0.02). The gametogenetic cycle followed the increase in seawater temperatures, and reached peak values during the warmest months of the year (Fig. 5). Mean chlorophyll-a concentrations and photoperiod had no correlation with GI (Spearman rank order coefficient, GI_W:_ *r*_s _= −0.21, p = 0.48 and *r*_s _= −0.08, p = 0.81; GI_D:_ *r*_s _= 0.13, p = 0.67 and *r*_s _= −0.43, p = 0.14, respectively). Nonetheless, the annual fluctuations in chlorophyll-a concentrations showed continuous increase in chlorophyll-a starting by the end of October, following the spawning event (Fig. 5), reaching its annual high in the following months. In turn, the annual peak of the photoperiod cycle in June coincided with the first significant increase in gonad index of the annual reproductive cycle, immediately followed by the first appearance of mature gonads (in July) in both sexes (Fig. 4).

### Oocyte growth and maturation

The monthly diameters of oocytes and ova are given in Fig. 6 and Supplementary Fig. S3 (illustrated as size frequency distributions). Oocytes were visible at basal levels throughout the year while ova were recorded from July through October. Oocyte mean diameters varied significantly between months (permutation ANOVA, *df* = 12, p < 2.2e-16) as they gradually increased in size from April to October, followed by a sharp decrease in oocyte diameter in November (Fig. 6). Ova mean diameters also varied significantly between months (permutation ANOVA, *df* = 3, p < 2.2e-16), reaching their annual maximum in September to October and were absent from all samples in the consecutive month. As ova did not increase in size after reaching maturation, monthly differences in mean ova diameters were the result of oocytes growing larger later in the season. This pattern coincided with the observed gametogenetic cycle and subsequent spawning.

## Discussion

### Body size and sex ratios

Although some sea urchins show external sexual dimorphism^29^, no external sexual characteristics have been reported in *Diadema*^1^. Similarly, in the current study, no external sexual dimorphism or variation in texture and colouration of the gonads between males and females were evident in more than 670 specimens examined. Furthermore, no size differences were observed between males and females *D. setosum* in allometric comparisons of populations from the GOA (Eilat) and WIO (Zanzibar) (Fig. 2).

*Diadema* are gonochoric with only rare reports of hermaphroditism^29^. In line with previous observations, all specimens in the current study had separate sexes. Sex ratios in echinoids have been predominantly reported to be 1:1^11^, although a few reports showed deviation from this ratio. When deviations occur, however, they rarely stray vastly from the 1:1 ratio. *D. setosum* is no exception^1^ and most reported populations have a 1:1 ratio^1^,^17^,^30^, although reports of slight deviations do exist (Hori *et al*.^31^ found more males than females (1:0.7) in 459 *D. setosum* individuals from Singapore). *D. setosum* sex ratios in the current study deviated significantly from a 1:1 ratio in the GOA (1:0.59 females to males, in 360 specimens sampled), whereas *D. setosum* populations from Zanzibar did not (n = 414), matching the situation in adjacent populations from Kenya (Muthiga, unpubl. in Muthiga and McClanahan^1^). The high female proportion observed in the GOA throughout the duration of this study is unusual. Sex determination mechanisms in echinoids (as well as all other echinoderms) are, however, largely unknown^1^,^29^, and the causes for unequal sex ratios are currently unidentified. Nonetheless, extreme environmental conditions such as unusually cold winter temperatures^29^ and parasitic infection by ascothoracid crustaceans have previously been suggested as possible explanations (J.S. Pearse and P. Newell, personal communication in Coppard and Campbell^14^). In Fiji, high levels of tributyltin (TBT) in the seawater have been suggested as the cause of extremely femininely biased *D. setosum* populations^14^. As no parasitic infections have been observed in specimens from the current study this explanations seem unlikely for the GOA populations. High TBT concentrations, on the other hand, have been measured in the past from sites adjacent to the current sampling locality^32^. Interestingly, sex ratios in *Echinometra* sampled in the same locality on the exact same dates, showed equal proportions of males and females^33^ – possibly indicating differential susceptibility to pollutants. Specimens of *Tripneustes gratilla elatensis*, again from the same sampling site, showed mass skeletal deformations that are thought to have been caused by chemical pollutants^34^, while other echinoid species in the area, including *Diadema* and *Echinometra*, showed no signs of deformations. Evidently, different echinoid species are susceptible to different extents by varying environmental perturbations, most likely owing to their different life histories, nutrition and microhabitat distribution. Although more studies are needed to elucidate the effect of environmental conditions on echinoid sex determination, instances of skewed sex ratios may serve as indication for potential detrimental processes that may prevail in the environment. The abundance of stressors to the marine environment in the GOA^24^ calls for special attention for any deviation from expected values or violation of equilibriums.

### Gametogenesis – evidence from histology and gonad indexes

Histological analysis of the gonads of *Diadema* from the GOA revealed that gametogenesis in both sexes was highly synchronised with mature individuals appearing from July to October. Interestingly, the mature phase of female *Diadema* in the GOA (July to October) seemed to be twice as long as that of males (July to August) (Fig. 4). Although sufficient to indicate synchronous spawning, these non-completely overlapping spawning periodicities are perplexing. It is possible that male spawning periodicities are underestimated in the current study as the number of sampled females always exceeded the number of males (up to 2.5:1), consequently better representing intra-population diversity in females. Alternatively, an extended spawning period by only one of the sexes have also been claimed to be an adaptive strategy to enhance fertilisation success by ensuring fertilisation of individuals (in this case males) spawning late or out of season^25^,^35^.

Gonad indexes (GI), the ratio between gonad and body size, have long been used as a tool for studying the reproductive cycle of a large range of species^19^ including many echinoids^12^,^36^. In principle, the GI is applied to depict temporal variations in gonad size that reflect the phases of the reproductive cycle^19^. However, many concerns and criticisms have been stressed regarding the validity of GI and its implication in reproductive studies^37^,^38^. Two fundamental drawbacks are associated with the use of gonad indexes: 1) they provide no information on the cellular level within the gonads, thus, when nutritive phagocytes within the gonads are used for synthesis of gametes, the index may remain the same despite the progress in gametogenesis^19^,^39^. 2) Most indexes implicitly assume an isometric relationship between gonad and body size that is, in most cases, not verified and in some instances utterly wrong^37^. In the current study, we have used histological analyses as well as several gonadal indexes and comparisons with ANCOVA to mitigate the risk of drawing erroneous conclusions. Indeed, our GI analyses (in all of the indexes used) demonstrated an increase in the index during November and December (Supplementary Fig. S1). However, our histological data clearly shows that no mature individuals and no ripe gonads were present past October, refuting the possibility of a second spawning event in the observed population. In fact, the high index values in November and December were predominantly recorded in recovering individuals (Fig. 4). Other causes than reproduction can potentially lead to changes in gonadal reserves and manifest as peaks in the GI^40^. King *et al*.^41^ for example, noticed that the gonads of *Centrostephanus rodgersii* returned to the recovering stage within a month of spawning with the GI returning to near pre-spawning levels. Similarly, post-spawning growth in *Diadema* from the GOA also appears to occur rapidly as the spent phase was encountered in relatively low frequencies. Rapid post-spawning growth was also reported in other echinoid species such as *C. rodgersii*^41^, *Strongylocentrotus franciscans*^42^ and *Paracentrotus lividus*^36^. Guillou and Michel^43^ attributed the occurrence of such GI peaks to abnormally low seawater temperatures in *Sphaerechinus granularis* off south Brittany. Seawater temperatures abnormalities may also be driving the currently observed post-spawning increase in GI for *D. setosum* from the GOA, as 2010 was significantly warmer than other years, and in fact had the warmest winter on record since measurements began by the Monitoring Program at the Gulf of Eilat (Israel National Monitoring Program at the Gulf of Eilat, 2014 annual report; Fig. E3).

Small, pre-vitellogenetic oocytes were present in the ovaries throughout the year (Fig. 6, Supplementary Fig. S3). Ovaries began accumulating mature ova as early as July and had attained high ova content by September, prior to spawning, when oogenesis was completed. However, similar to other echinoid species, not all oocytes mature to ova, and excess oocytes undergo phagocytosis, facilitating the reallocation of nutrients to the remaining growing oocytes^10^,^27^. Production of a large number of excess oocytes consequently leads to phagocytosis of the unspawned portion, producing a bimodal oocytes size frequency distribution^25^. In contrast, when only a low number of excess oocytes are being produced, the continuous progression of small to large oocytes is manifested as a unimodal distribution^33^,^35^. In this respect, *Diadema* from the GOA seem to produce few excess oocytes per gametogenetic cycle and their spawning is most likely exhaustive.

### Geographic patterns of *Diadema* reproduction

**Figure 1:**
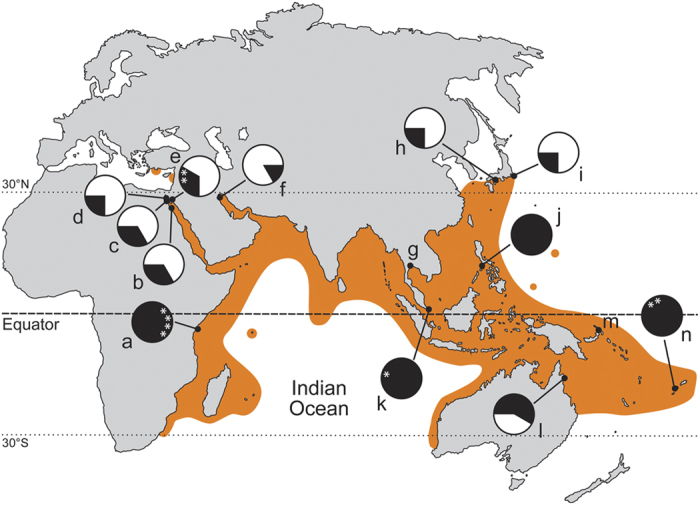
Distribution map and spawning periodicities of *Diadema setosum*. Locations of known spawning times are indicated by dots. Radial plots indicate estimated spawning periods (black portions) for each location. The plots are constructed of 12 equally sized slices representing the monthly annual cycle from January to December (clockwise). In sites of continuous year-round spawning, peak spawning months are indicated by asterisk (if such distinction was mentioned in the literature). Distribution estimates were based on: Pearse^12^,^57^; Clark and Rowe^58^; Marsh and Marshall^59^; Rowe and Gates^60^; Shin^61^; Lessios *et al*.^4^; James^62^; Sastry^63^; Yokes and Galil^64^; Nader and El Indary^65^ as well as data available from the OBIS website (http://www.iobis.org/home). Lowercase letters indicate the underlying publication of spawning periodicities for the particular location (only publications specifically referring to spawning periodicities were considered for the construction of this map). a: Kenya - Muthiga^18^; b: Al-Ghardaqa - Pearse^10^; c: Wadi-el-Dom (Gulf of Suez)- Pearse^10^; d: Port Tawtiq (Gulf of Suez) - Fox^46^; e: Eilat (Gulf of Aqaba)- this study; f: Kuwait - Alsaffar and Lone^17^; g: Sichang (Thailand)- Kobayashi^45^ (report of spawning in a two month study during February and March) ; h: Seto (Japan)- Onoda^66^, Kobayashi and Nakamura^67^; i: Misaki (Japan)- Yoshida^30^; j: Philippines - Tuason and Gomez^44^; k: Singapore - Hori *et al*.^31^; l: Low Isles (Great Barrier Reef) - Stephenson^68^; m: Rabaul (New Britain Island) - Pearse^12^ (report of spawning by field observations on 24.02.1965 based on pers. comm. from J. J. Gonor); n: Fiji - Coppard and Campbell^14^. The map was created based on the Wikimedia Commons public domain map file *BlankMap-World6.svg* (https://commons.wikimedia.org/wiki/File:BlankMap-World6.svg) and manually edited using CorelDRAW x4.

**Figure 2:**
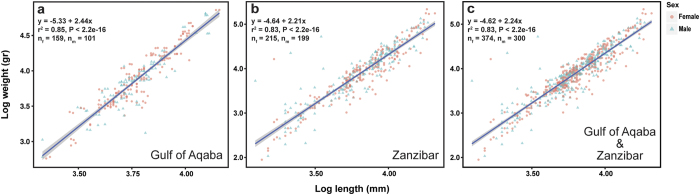
Size and sex relationships in populations of *Diadema setosum* from the Red Sea (Eilat) and Western Indian Ocean (Zanzibar). Linear regression models of diameter and weight (log transformed) in two populations of *D. setosum*: (**a**) Eilat, (**b**) Zanzibar, and (**c**) the pooled data from both locations. Females are denoted by red circles; males by green triangles. Regression lines with 95% confidence interval are fitted for each plot and the corresponding equations and respective r^2^ and p-values are provided. The number of samples used, for both females (n_f_) and males (n_m_), is given below the corresponding equations.

**Figure 3:**
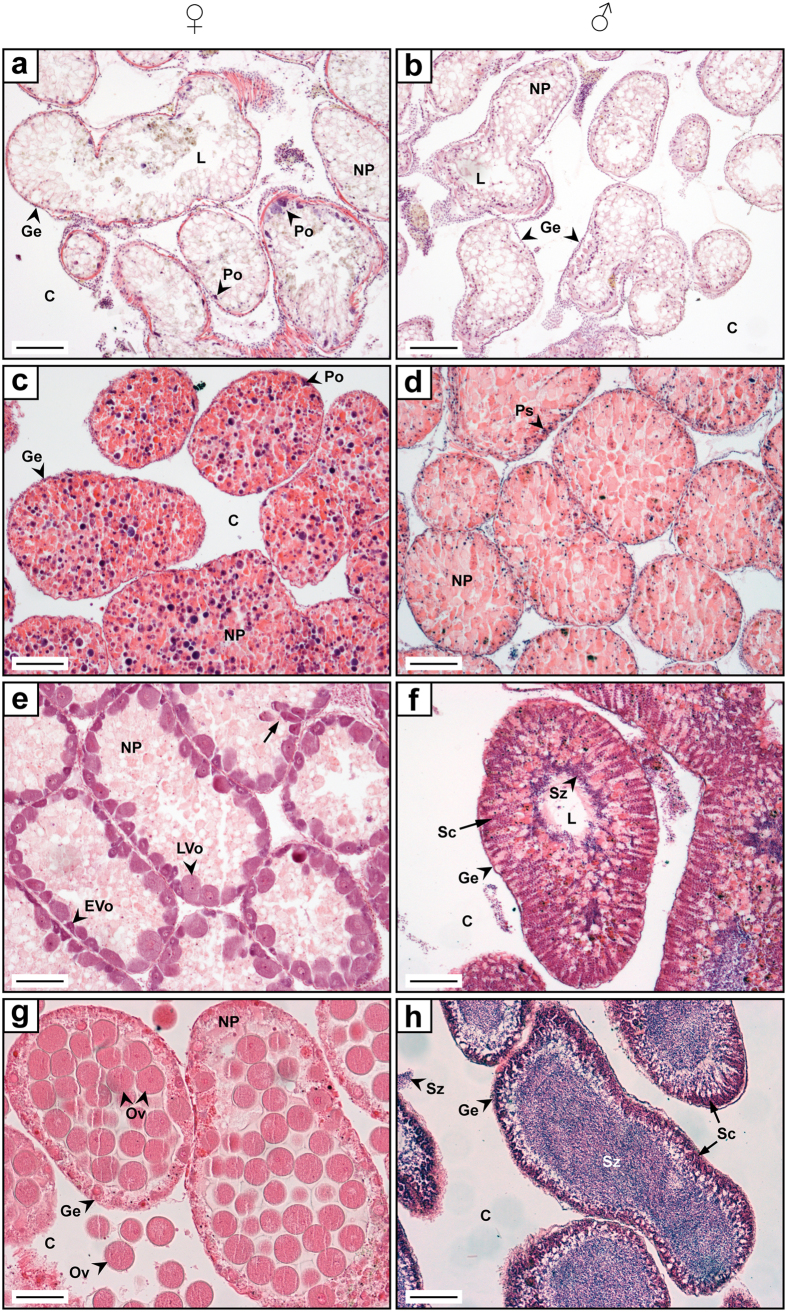
The reproductive stages of *Diadema setosum* from the Gulf of Aqaba. Histological photomicrographs of ovaries **(a,c,e,g)** and testes **(b,d,f,h)**. Cross-sections through acini representing reproductive stages I–IV. Stage I (spent): Gonads are largely devoid of contents showing ova-free lumen in females **(a)**, and spermatozoan-free lumen in males **(b)** and may contain unspawned ova and spermatozoa undergoing lysis. A thin layer of NPs is present along the ascinal walls in both sexes and may form a pale meshwork across the ascinus. Strongly basophilic previtellogenetic oocytes or primary spermatocytes, staining dark purple with Hematoxylin and eosin, may be present along the ascinal wall. Stage II (recovering): NPs proliferate throughout the gonads from the ascinal wall to the centre, gradually filling the lumen of ovaries **(c)** and testis **(d)**. Limited groups of primary spermatocytes and clusters of previtellogenetic oocytes start appearing in the testicular and ovarian germinal epithelia, respectively, and may occasionally project centrally. Stage III (growing): With the onset of vitellogenesis oocytes grow in size and become decreasingly basophilic. Both early and late vitellogenetic oocytes may be present along the ovarian wall and gradually migrate to the ovarian lumen as they mature (indicated by arrow) **(e)**. All stages of germ cells are evident in the male germinal epithelium and continuously increase in number as new spermatogonia develop basally while spermatocytes migrate to the testicular lumen, where they accumulate as mature spermatozoa, forming visible columns of darkly stained cells **(f)**. NP deplete and progressively occupy less space in both males and females. Stage IV (mature): By the end of this stage the NP layer in both ovaries and testes is largely exhausted. Ovaries are packed with mature ova, while oocytes at different maturation stages may still be evident in the germinal epithelium **(g)**. The testicular lumen is densely packed with spermatozoa. Occasionally some ova and spermatozoa may be evident in the coelom **(h)**. Scale bars represent 100 μm. **Ge** germinal epithelium; **C** coelom; **Po** previtellogenetic oocyte; **EVo** early vitellogenetic oocyte; **LVo** late vitellogenetic oocyte; **NP** nutritive phagocytes; **Ov** ova; **L** lumen; **Sc** spermatocytes; **Sz** spermatozoa; **Ps** primary spermatocytes.

The currently observed pattern of *D. setosum* summer restricted reproduction in the GOA, at the north-western edge of its range, lends support to the ‘equatorial model’ of Giese and Pearse^19^. *D. setosum* continuous reproduction around the tropics was recorded from as widely distant localities as: Kenya^18^, Singapore^31^, the Philippines^44^, and Fiji^14^ (Fig. 1). Reports from other locations such as Thailand^45^ and Rabaul in the New Britain Islands^12^, where spawning has been reported to occur in February and March, did not cover the entire annual cycle and reproduction in these areas may in fact still reflect a continuous pattern.

**Figure 4:**
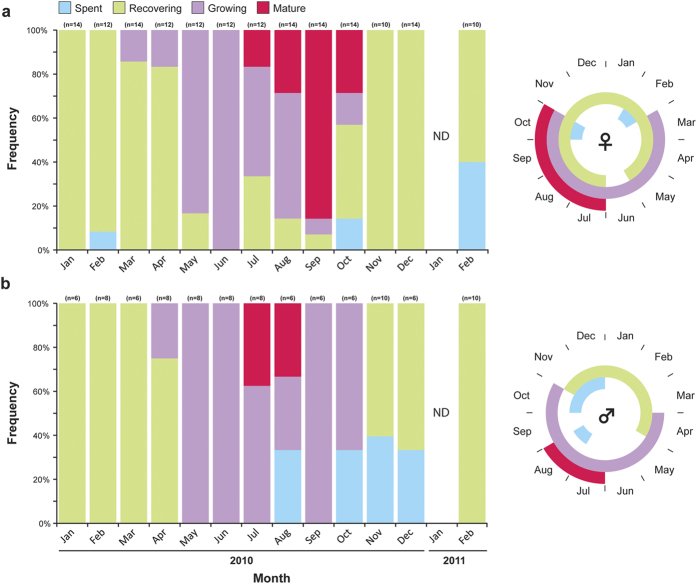
The annual gametogenetic cycle of *Diadema setosum* from Eilat (Gulf of Aqaba). The relative frequencies (%) of gonad developmental stages in monthly samples of **(a)** females and **(b)** males as defined by histological cross-sections. Frequencies are based on histological analysis of 20 specimen per month studied from January 2010 to February 2011. Colours indicate reproductive stages I–IV (corresponding to stages: Spent, Recovering, Growing and Mature, respectively) as defined in the text (see Fig. 3 for detail). ND corresponds to no data for that sampling month. Radial schematic plots provide a graphical representation of the transition and overlap of the different reproductive stages based on a monthly annual cycle.

**Figure 5:**
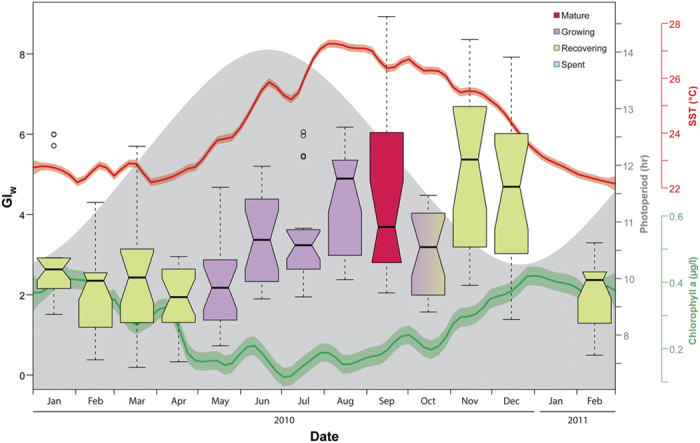
Temporal patterns of environmental gradients during the reproductive cycle *Diadema setosum*. Monthly gonad index (GI_W_) calculated as wet gonad weights/total wet body weight × 100 of *Diadema setosum* from the GOA and monthly gradients of selected environmental variables. Boxes represent monthly GI_W_; centre black lines show the medians; box limits indicate the 25^th^ and 75^th^ percentiles; the 95% confidence interval of each median is represented by the notches and is defined as +/−1.58 × IQR/sqrt(n) (with n representing the monthly number of samples); whiskers extend to minimum and maximum values with open circles representing outliers; width of the boxes is proportional to the square root of the sample size. Indexes calculated based on 20 specimens per month. No data are available for January 2011. The colour of the boxplots corresponds to the dominant reproductive stage of that month (see Fig. 4 for details). Grey zone illustrates photoperiod. Red line illustrates daily measured sea surface temperatures (°C) and green line illustrates daily measured chlorophyll-a concentrations (μg/l) fitted as a smooth curve (solid lines) and standard errors (shaded margins). The smooth was calculated by local polynominal regressions.

In contrast to *Echinometra*, where striking differences were observed between GOS and RSMB populations (the former showing seasonal reproduction while the latter continuous^27^), no seasonal reproductive differences between these basins were observed in *Diadema*^10^. In the GOA the spawning season of *D. setosum* is restricted to the warm summer months (Fig. 1e), similar to the spawning season reported for *D. setosum* from both the GOS and RSMB^10^,^46^, with peak spawning occurring between September and October. This is consistent with *D. setosum* reports from all but one locality throughout the northern hemisphere (Fig. 1). Alsaffar and Lone^17^ reported peak spawning between April and May off the coast of Kuwait at the northern tip of the Persian Gulf. This deviation in the spawning season despite similar latitudinal position with the GOA and GOS may be attributed to the unique environmental conditions in the Persian Gulf. The area shows extreme seasonal differences in seawater temperatures, varying from a minimum of 13.2 °C to a maximum of 31.5 °C^47^. More so, Alsaffar and Lone^17^ reported fluctuations from a minimum of 10.6 °C in January and a maximum 32.8 °C in August throughout the duration of their experiment. Such extreme high summer temperatures may be exceeding the maximum threshold for *Diadema* reproduction, shifting spawning periodicities to cooler months in the northern Persian Gulf. Indeed, the seawater temperatures during the spawning season of the Kuwaiti populations (24–30 °C, Apr-May) are comparable with the spawning season temperatures at the GOS (25–30 °C, Jun-Sep) and GOA (25–28 °C, Jul-Oct). Furthermore, Pearse^10^ notion of *D. setosum* being reproductively inactive at SST below about 25 °C seems to hold true also for the GOA population, as despite of the fact that growing stage individuals were already observed in May, a significant increase in gonad growth only occurred in June once SST crossed the 25 °C threshold (Fig. 4).

**Figure 6:**
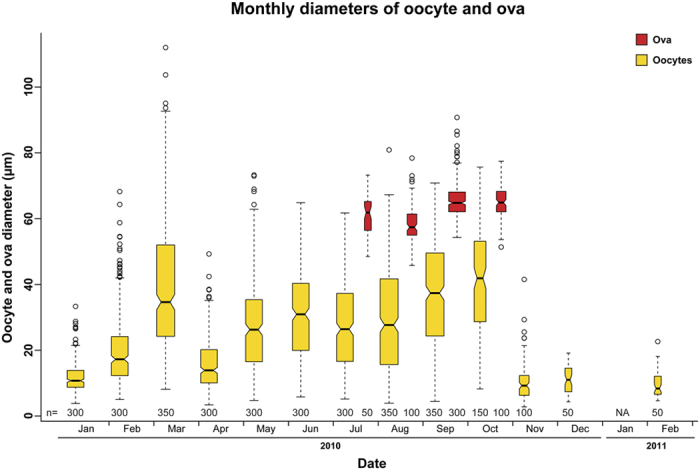
Temporal variation in ova and oocyte diameters. Diameters (μm) of ova (red boxes) and oocytes (gold boxes) from female *Diadema setosum* from the GOA. Measurements conducted from January 2010 through February 2011. Boxes represent monthly average oocyte diameters; centre black lines show the medians; box limits indicate the 25^th^ and 75^th^ percentiles; the 95% confidence interval of each median is represented by the notches and is defined as ±1.58 × IQR/sqrt(n) (with n representing the number of samples as indicated under the boxes); whiskers extend to minimum and maximum values with open circles representing outliers; width of the boxes is proportional to the square root of the sample size.

The latitudinal similarity of the latter three localities may also provide insights into the role of photoperiod in regulating *Diadema* reproduction. In contrast to temperature that varies considerably between these three localities for any given point in time, photoperiod, being governed solely by latitude, does not. Thus, although photoperiod has been noted as one of the main exogenous factors that control the reproductive cycle in echinoids^48^,^49^,^50^, it cannot account for the currently observed differences in spawning periodicities. Similarly, even within the GOA, no correlation was found between GI and photoperiod (Fig. 6). These results appear to be in agreement with Pearse^27^, who found no such correlation in *Echinometra* populations in the adjacent GOS. Nonetheless, the longest mean day length was reached in June, corresponding with a significant increase in GI (Fig. 6). Thus, although photoperiod does not seem to be the trigger for spawning, shortening days may still serve as an exogenous cue for gametogenesis. A similar tendency of gametogenesis initiation triggered by the onset of shortening day length was also reported in other echinoid species, such as *Strongylocentrotus droebachiensis*^49^, *Eucidaris tribuloides*^51^, as well as *Echinometra* sp. from the GOA^33^.

Similar to photoperiod, no direct correlation was found between *D. setosum*’s reproductive cycle and chlorophyll-a concentrations that is used as a proxy for phytoplankton abundance. Although some echinoid spawning periodicities have been associated with phytoplankton blooms^52^, these are unlikely to directly entrain reproductive cycles due to their huge spatial variability^11^. Food availability is nonetheless an important factor for the developing larvae and thus spawning periodicities timed during or just prior to annual peaks in phytoplankton are advantageous for the newly developing larvae, as the latter are ensured a constant supply of food throughout the early stages of their lives. In the GOA, *D. setosum* spawn at the onset of to the annual increase in chlorophyll-a concentrations. A similar pattern was also observed in *Echinometra* from the same localities^33^ as well as in Kenya^35^.

Indeed, the reproductive cycle of the two most abundant and ecologically significant echinoids in the GOA - *Echinometra* sp. and *D. setosum*^21^ – largely overlaps. Although gametogenesis in *Diadema* appears to be longer than in *Echinometra*, peak spawning of these two species occurs between September and October, with the reproductive season of *Echinometra* lasting almost twice as long as that of *Diadema*^33^. One possible explanation for this difference may be attributed to the different life histories and behaviour of these two species. As *Diadema* are considerably more mobile than *Echinometr*a^53^ and often display aggregative reproductive behaviour^8^, an individual may improve its reproductive success by grouping with other conspecifics and tightly timing its reproductive effort to a short, exhaustive and highly synchronised spawning event. In contrast, *Echinometra* refrains from straying away from their crevices, and has never been observed to aggregate prior to spawning. In populations of relatively low densities, such as in the GOA (Bronstein unpubl. data), *Echinometra* may benefit from an extended spawning season during which local groups in the population, defined by their spatial distribution, may spawn at different times during the season, triggered by the spawning of their nearby individuals.

The current study is the first to demonstrate the reproductive cycle of *D. setosum* in the Gulf of Aqaba. It facilitates a geographical comparison with other localities of reported *D. setosum* reproduction and enables evaluation of the prevailing environmental cues that control reproduction in echinoids. In addition, the current study highlights potential pitfalls in the use of gonad indexes and the risk of drawing erroneous conclusions when GI data is not supported by histological analyses. Further studies of these important reef structuring echinoids are needed to facilitate comparisons over multi-annual cycles and elucidate the forces that drive biased sex ratio in echinoids.

## Methods

Twenty individuals of *Diadema setosum* were collected monthly along the coast of Eilat in the GOA (Red Sea, 29°32′48.1″N, 34°57′12.2″E; see Fig. 1) between January and December 2010, with an additional sampling conducted in February 2011. The samples were collected haphazardly by snorkelling at depths of 1 to 3 m and brought to the laboratory at the Inter-University Institute (IUI) in Eilat for further analysis.

The largest corona diameter (measured at the ambitus), corona height, and wet weight of each specimen were recorded. Measurements to the nearest 0.5 mm were performed using thin-blade Vernier callipers to prevent interference by the spines. Weight was measured to the nearest 0.001 g after drying each specimen from excessive water for 5 min. After weighing and completion of external measurements, specimens were dissected and the gonads removed and weighed to the nearest 0.001 g (wet weight). Sex was determined by observing a small piece of gonad under a light microscope and later confirmed using the histological sections. Samples from Zanzibar (06°01′20.99″S, 39°25′30.21″E) in the western Indian Ocean were added to the analyses to facilitate comparison with other *Diadema* populations. In total 260 and 414 specimens were analysed from Eilat and Zanzibar, respectively.

Following external and gonadal measurements, gonad samples were prepared for histological examination. Preparation, documentation and analyses of the histological sections follow Bronstein and Loya^33^. Male and female gonads were classified into four gametogenetic stages, adopted from the staging methods of Yoshida^30^ and Pearse^10^, corresponding to fluctuations in both the nutritive phagocyte (NP) and germ cell populations. Temporal variations in oocyte and ova diameters were assessed by randomly measuring 50 oocytes and/or ova of selected female specimens. Only primary oocytes sectioned through the nucleolus and ova sectioned through the nucleus were considered for this analysis.

Sea surface temperatures (SST) and chlorophyll-a measurements were obtained from the Israel National Monitoring Program at the Gulf of Eilat (http://www.meteo-tech.co.il/eilat-yam/eilat_periodical_en.asp). Photoperiod data were obtained from the Earth System Research Laboratory of NOAA Global Monitoring Division (http://www.esrl.noaa.gov/gmd/grad/solcalc/calcdetails.html).

Data analyses were performed using R statistical software^54^. When data violated test assumptions of normal distribution and homoscedasticity, non-parametric tests or permutation analysis were performed. Permutations were performed using the packages *lmPerm* (Wheeler 2010; lmPerm: Permutation tests for linear models. http://www2.uaem.mx/r-mirror/web/packages/lmPerm/lmPerm.pdf), allowing all permutations of Y (i.e., Perm = “Exact”). Chi-squared tests were used to test possible differences in sex ratios. Due to the problems associated with gonad indexes^37^,^55^, several indexes have been generated and compared to explore monthly differences in gonad development. Variation in gonad weight was additionally examined by performing permutation analysis of co-variance (ANCOVA) (with gonad wet weight as the response variable and total wet weight or test diameter as the covariates). Initially, Mann-Whitney *U* tests were applied to test for between-sexes differences in every month (p-values corrected for multiple testing using the False Discovery Rate (FDR) correction). As no significant differences were found, both sexes were pooled for further analyses. Gonad indexes (GI) were created based on measurements of gonads wet weights, test diameters, and total wet weights. Initially, GI_W_ (gonad index wet weight) was calculated, as this is the most commonly used gonadosomatic index^37^. This index is based on gonad wet weight and the total wet weight (GI_W_ = Gonad wet weight (g)/Total wet weight (g) × 100). However, as some sea urchin species may lose coelomic fluid once removed from the field in a process that varies among individuals^56^, test diameters have been used as an alternative to total wet weight, producing GI_D_ (GI_D_ = Gonad wet weight (g)/Test diameter (mm) × 100). Still, as both former indexes implicitly assume an isometric relationship between the two measurements (i.e., that both the gonad and body weight/diameter increase at the same rate), a third, standardised index (SGI) has been implemented following Ouréns *et al*.^38^: SGI_i_ = logGWW_i_ − logGWW_i_; where logGWW_i_ represents the logarithm of the observed gonad wet weight in the *i*th individual, and logGWW_i_ is the predicted value from the regression for an individual of this body size. Kruskal-Wallis non-parametric analysis of variance (ANOVA), followed by Dunn’s post-hoc tests, were used to check for differences in the calculated indexes between months using the R package *dunn.test* (Dinno 2016; Dunn’s Test of Multiple Comparisons Using Rank Sums. https://cran.r-project.org/web/packages/dunn.test/dunn.test.pdf). Monthly mean GI values of the different indexes calculated were tested against monthly mean SST, chlorophyll-a, and photoperiod using Spearman’s rank correlation. Pairwise Kolmogorov-Smirnov tests were applied to test for monthly differences in size frequency distributions between oocytes and ova and p-values were adjusted for multiple testing to minimise false discovery rate, using the Bonferroni correction. Monthly mean diameters of oocytes and ova were compared using permutation ANOVAs followed by Tukey’s honest significant difference (HSD) post-hoc test.

## Additional Information

**How to cite this article**: Bronstein, O. *et al*. Reproduction of the long-spined sea urchin *Diadema setosum* in the Gulf of Aqaba - implications for the use of gonad-indexes. *Sci. Rep.* **6**, 29569; doi: 10.1038/srep29569 (2016).

## Supplementary Material

Supplementary Information
