# Supplementary material for: Reproduction of the long-spined sea urchin Diadema setosum in the Gulf of Aqaba - implications for the use of gonad-indexes
Source: Sci Rep. 2016 Jul 12;6:29569. doi: 10.1038/srep29569 (PMC4941735; doi:10.1038/srep29569)

Supplementary Information

Reproduction of the long-spined sea urchin *Diadema setosum* in the Gulf of Aqaba - implications for the use of gonad indexes

Authors

Omri Bronstein*[,a,b]

Andreas Kroh[a]

Yossi Loya[c]

Affiliation

[a] Natural History Museum Vienna, Geological-Paleontological Department, Burgring 7, 1010 Vienna, Austria

[b] The Steinhardt Museum of Natural History, Israel National Centr for Biodiversity Studies, Tel-Aviv University, Tel-Aviv 69978, Israel

[c] Department of Zoology, The George S. Wise Faculty of Life Sciences, Tel-Aviv University, Tel-Aviv 69978, Israel

* Corresponding author

E-mail address:

omribronstein@gmail.com
Tel: +43-1-52177-610
Fax: +43-1-52177-459

Supplementary Information

Figure S1. Temporal variations in *Diadema setosum* gonad indexes. (a) Boxes represent monthly SGI, GI_D and GI_W(see text for calculations of the different indexes); center black lines show the medians; box limits indicate the 25th and 75th percentiles; the 95% confidence interval of each median is represented by the notches and is defined as +/- 1.58*IQR/sqrt(n) (with n representing the monthly number of samples); whiskers extend to minimum and maximum values with open circles representing outliers; width of the boxes is proportional to the square root of the sample size Indexes calculated based on 20 specimens per month. **(b)** Plots represent monthly SGI, GI_D and GI_W (see text for calculations of the different indexes). Smoothed curves were added (solid lines) and standard errors (grey shadings) were fitted to the data. The smooth was calculated by local polynomial regressions. No data are available for January 2011.

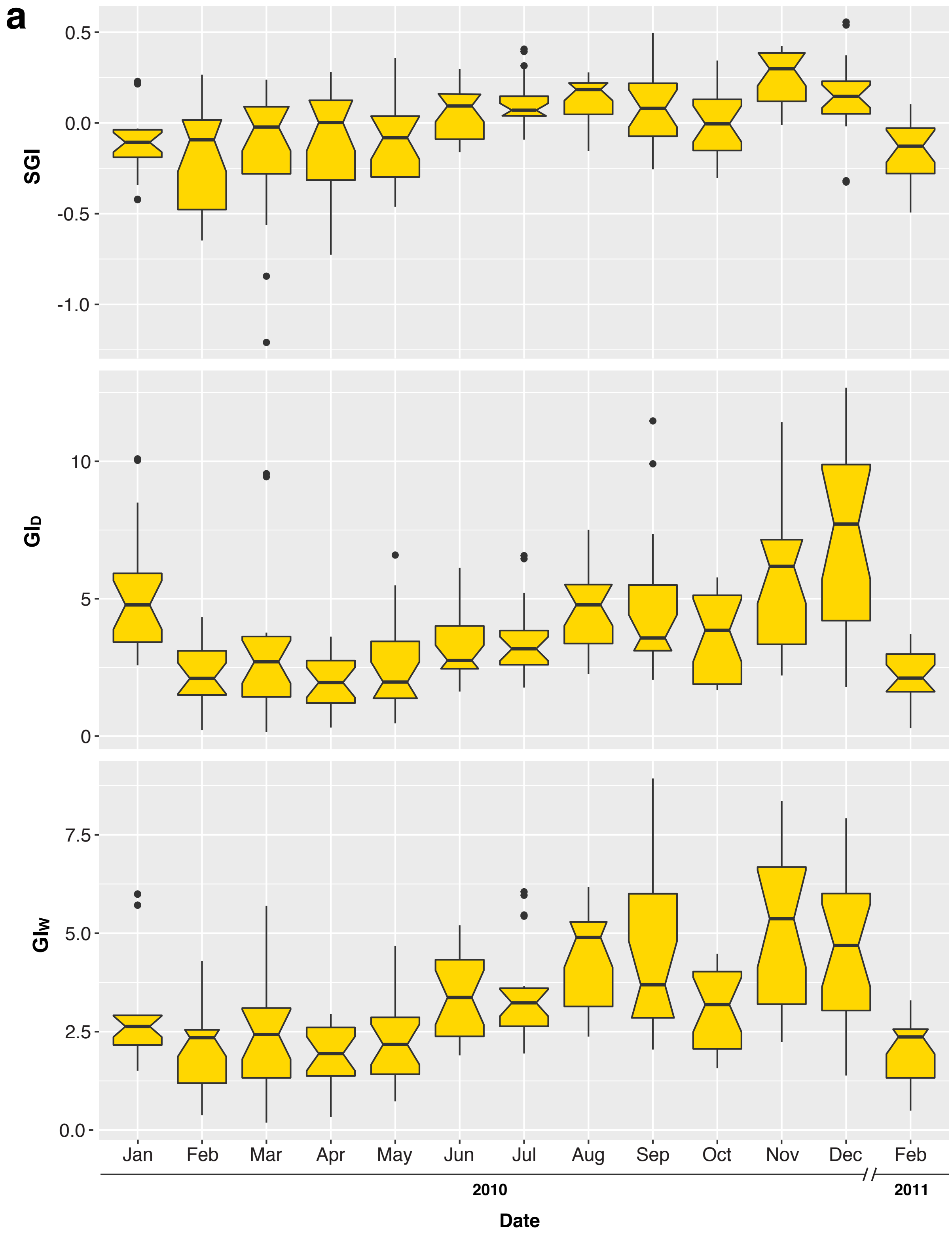

Supplementary Information

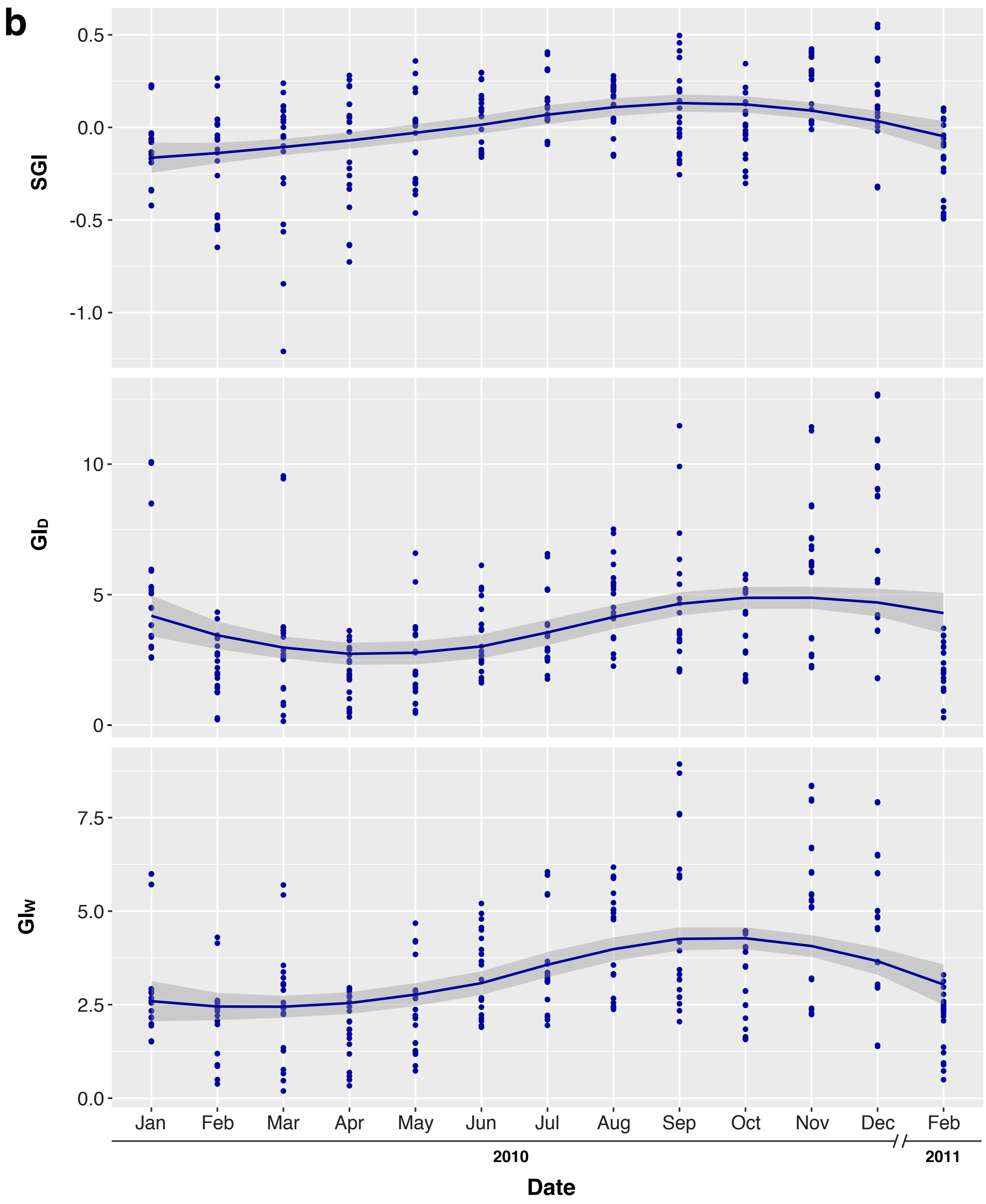

Supplementary Information

Figure S2. Slope pattern of gonad and body measurement regressions. Monthly changes of the allometric exponent β with analyses of test diameter or total wet body weight as covariates. Red line represents β for test diameter and blue line for total wet body weight.

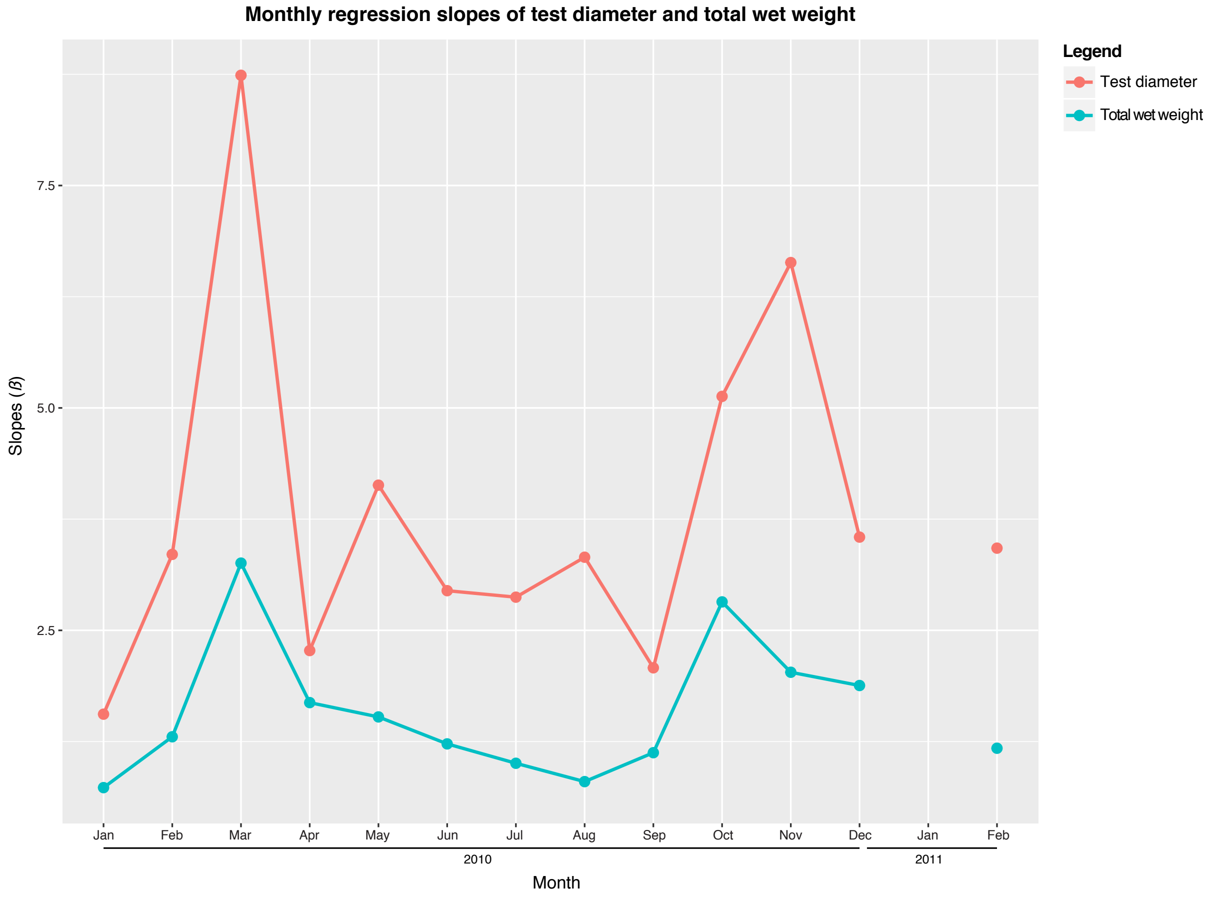

Supplementary Information

Figure S3. Size distribution of ova (red polygons) and oocytes (yellow polygons) of female *Diadema setosum* from the GOA from January 2010 to February 2011. Oocyte and ova diameters (μm) were obtained from measurements of 50 random cells each, in all available female specimens per month.

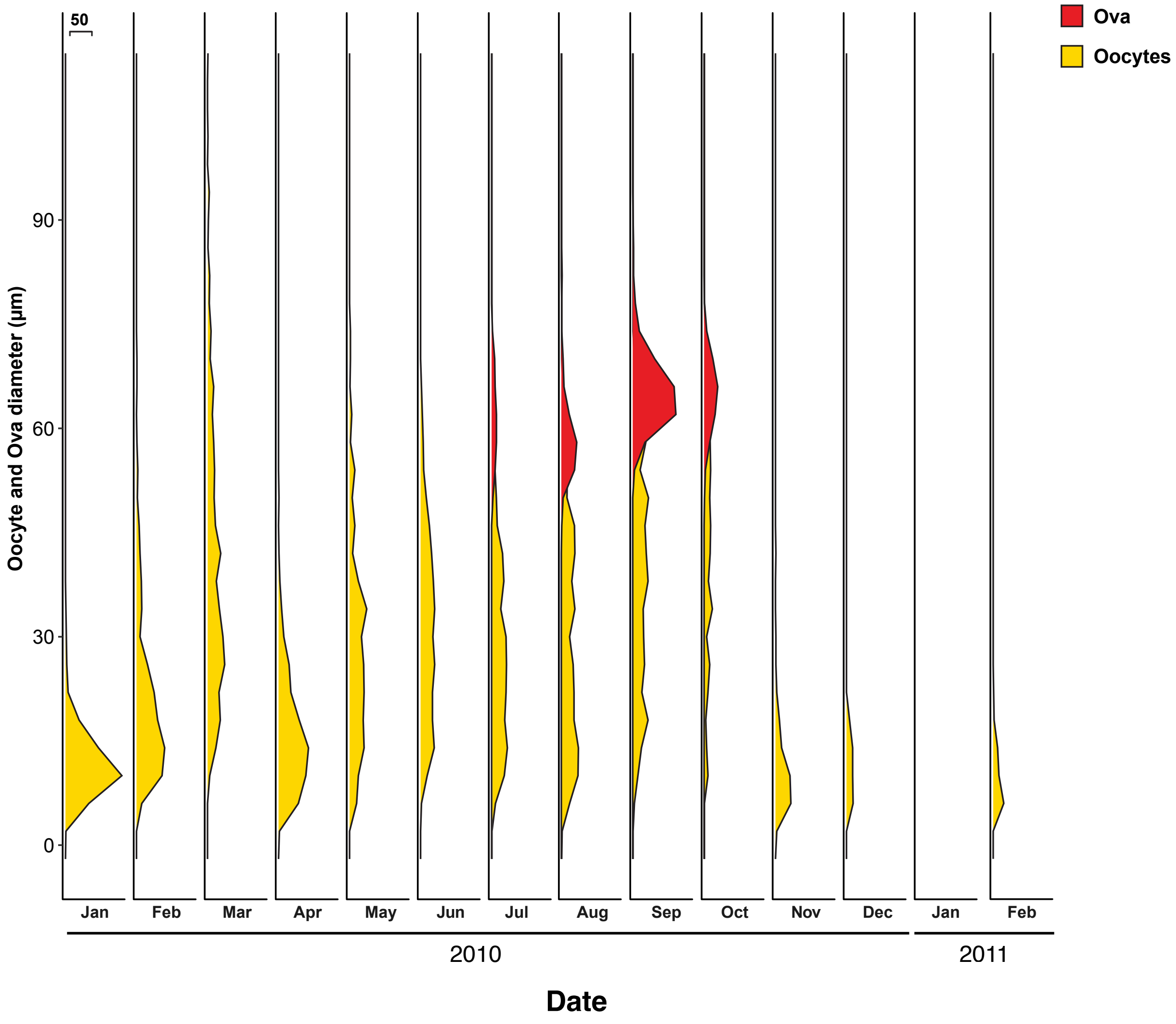

Supplement: Supplementary Information [file srep29569-s1.pdf]
